# Outcome research on esophagectomy analyzed using nationwide databases in Japan: evidences generated from real-world data

**DOI:** 10.1007/s10388-024-01080-w

**Published:** 2024-08-19

**Authors:** Yoshihiro Kakeji, Hiroyuki Yamamoto, Masayuki Watanabe, Koji Kono, Hideki Ueno, Yuichiro Doki, Yuko Kitagawa, Hiroya Takeuchi, Ken Shirabe, Yasuyuki Seto

**Affiliations:** 1 Division of Gastrointestinal Surgery, Department of Surgery, Kobe University Graduate School of Medicine, 7-5-2 Kusunoki-cho, Chuo-ku,Kobe, 650-0017 Japan; 2 Department of Healthcare Quality Assessment Graduate School of Medicine, The University of Tokyo, Tokyo, Japan; 3 Department of Gastroenterological Surgery, Cancer Institute Hospital of Japanese Foundation for Cancer Research, Tokyo, Japan; 4 Department of Gastrointestinal Tract Surgery, Fukushima Medical University, Fukushima, Japan; 5 Department of Surgery, National Defense Medical College, Saitama, Japan; 6 Department of Gastroenterological Surgery, Osaka University Graduate School of Medicine, Osaka, Japan; 7 Department of Surgery, School of Medicine, Keio University, Tokyo, Japan; 8 Department of Surgery, Hamamatsu University School of Medicine, Shizuoka, Japan; 9 Department of General Surgical Science, Gunma University Graduate School of Medicine, Maebashi, Gunma Japan; 10 Department of Gastrointestinal Surgery, The University of Tokyo Graduate School of Medicine, Tokyo, Japan

**Keywords:** Esophagectomy, Morbidity, Mortality, NCD, DPC

## Abstract

Esophagectomy for esophageal cancer is a highly invasive gastrointestinal surgical procedure. The National Clinical Database (NCD) of Japan, initiated in 2011, has compiled real-world data on esophagectomy, one of nine major gastroenterological surgeries. This review examines outcomes after esophagectomy analyzed using the Japanese big databases. Certification systems by the Japanese Society of Gastroenterological Surgery (JSGS) and the Japan Esophageal Society (JES) have shown that institutional certification has a greater impact on short-term surgical outcomes than surgeon certification. Minimally invasive esophagectomy has emerged as a viable alternative to open esophagectomy, although careful patient selection is crucial, especially for elderly patients with advanced tumors. The NCD has significantly contributed to the assessment and enhancement of surgical quality and short-term outcomes, while studies based on Comprehensive Registry of Esophageal Cancer in Japan (CRECJ) have provided data on patient characteristics, treatments, and long-term outcomes. The JES has conducted various questionnaire-based retrospective clinical reviews in collaboration with authorized institutions certified by JES. The Diagnosis Procedure Combination (DPC) database provides administrative claims data including itemized prices for surgical, pharmaceutical, laboratory, and other inpatient services. Analyzing these nationwide databases can offer precise insights into surgical quality for esophageal cancer, potentially leading to improved treatment outcomes.

## Introduction

Esophageal cancer ranks seventh among the most common types of cancer worldwide and sixth among the leading causes of cancer death [1]. Esophagectomy is a highly invasive procedure for treating esophageal cancer. Recently, several nationwide databases have been organized and many clinical analyses using these databases have reported real-world data. The National Clinical Database (NCD) of Japan was established in 2010 with major support from the Japan Surgical Society (JSS), the Japanese Society of Gastroenterological Surgery (JSGS), and eight other professional surgical societies. The NCD incorporates a board certification system, with web-based data collection starting in 2011 [2–4]. The estimated coverages of NCD were reported to be 90–95% by comparison with regional government report data and medical charts [5], and the audit works verified the NCD’s data and found high accuracy of data entry [6]. As of February 2023, a total of 5664 institutions had enrolled and approximately 1.5 million cases have been registered annually [7]. Despite a decrease in 30-day and 90-day mortalities after esophagectomy, there has been an increase in severe postoperative complications, possibly due to an aging population with higher rates of preoperative comorbidities [8]. Efforts have been made to reduce these complications, and this review focuses on short-term outcomes after esophagectomy analyzed using NCD.

The Comprehensive Registry of Esophageal Cancer in Japan (CRECJ) is a sizable database of Japan Esophageal Society (JES). The characteristics of this unique database include both precise short-term outcomes and long-term survival data. Annually, the JES gathers information on patient profiles, treatment modalities, and outcomes [9–12]. In 2019, the data collection approach transitioned from electronic submissions to web-based data collection via the NCD [13–15].

There is another nationwide inpatient data in Japan, the Diagnosis Procedure Combination (DPC) database [16]. The DPC is a case-mix patient classification system in Japan that is linked with a lump-sum payment system. All the 82 academic hospitals (80 university hospitals, the National Cancer Centre and the National Cerebral and Cardiovascular Centre) are obliged to adopt the DPC system, but adoption by community hospitals is voluntary. The DPC database covers approximately 90% of all tertiary-care emergency hospitals, 44% of institutions certified by the JSS, and 80% of institutions certified by the Japanese Association for Infectious Diseases for training board specialists [16]. This database aggregates over 8 million cases annually through hospital administrative claims data and discharge abstracts from more than 1200 facilities. As of June 2024, the DPC database encompasses 1786 hospitals and 480,000 beds, representing 85% of all acute-care hospital beds in Japan [17].

We review topics on esophageal cancer surgery in Japan, particularly focusing on studies related to nationwide data collection systems, to provide an overview of our learnings and to gather insights for improving clinical outcomes in the future.

### Development of NCD risk models and feedback system

**Fig. 1:**
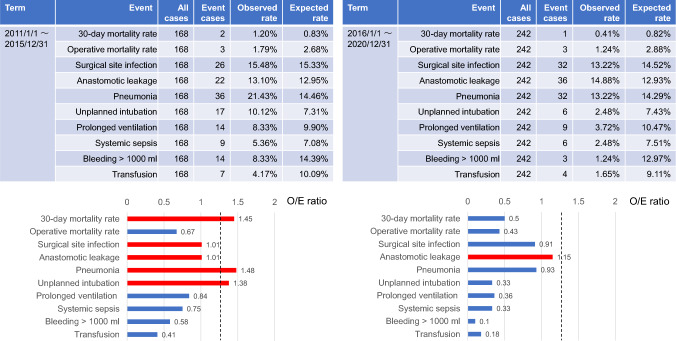
Facility’s severity-adjusted clinical performance (benchmark) compared with national data, illustrating short-term outcomes for a hypothetical facility. Upper tables: the NCD provide data on each facility’s severity-adjusted clinical performance (benchmark), which can be compared with national data (expected rate). Lower graphs: changes in outcomes pre- and post-implementation of operational procedures or perioperative management can be assessed through observed/expected (O/E) ratio comparisons

The NCD implements mortality and morbidity risk models for esophagectomy using preoperative variables to provide real-time feedback on predicted outcomes [18–20] (Table 1). This system aids healthcare professionals in treatment decision-making and obtaining informed consent from patients and their families. In addition, the NCD provides each facility’s severity-adjusted clinical performance, enabling comparisons with national data [2, 4]. Changes in outcomes pre- and post-implementation of operational procedures or perioperative management can be assessed through observed/expected (O/E) ratio comparisons (Fig. 1).

**Table 1:** Risk calculator for predictive mortalities and morbidities based on preoperative data input

Mortality or morbidity	Predictive rate%
30-day mortality rate	0.20
Operative mortality rate	1.80
Surgical site infection	15.20
Anastomotic leakage	11.50
Pneumonia	8.80
Unplanned intubation	8.50
Prolonged ventilation over 48 h	11.30
Bleeding > 1000 ml	15.10
Transfusion	25.60
Systemic sepsis	11.70

Table shows the predicted mortality rate and predicted complication rate for esophagectomy calculated using hypothetical patient’s data

### Nationwide studies on quality indicators and operative outcomes

#### Certification systems and outcomes

The Japanese Society of Gastroenterological Surgery (JSGS) instituted a board certification system in 1984, resulting in over 6000 surgeons becoming board-certified surgeons in gastroenterological surgery (BCS-Gs) since then. Konno et al. [21] assessed the correlation between the involvement of BCS-Gs and the surgical outcomes of gastroenterological surgeries. Their analysis revealed that 94.5% of esophagectomies were performed in hospitals with two or more BCS-Gs. A multivariable logistic regression model demonstrated that a higher number of BCS-Gs in hospitals was predictive of favorable surgical outcomes, particularly regarding operative mortality.

The JES introduced a certification system in 2009 for qualified surgeons as “Board Certified Esophageal Surgeons” (BCESs) and institutes as “Authorized Institutes for Board Certified Esophageal Surgeons” (AIBCESs). Motoyama et al. [22] utilized the NCD to investigate short-term outcomes following esophagectomy, considering the certifications statuses of surgeons and institutes. Their findings indicated a significantly lower incidence of anastomotic leakage with BCESs compared to Non-BCESs (13.2 vs. 14.0%). In addition, the overall surgery-related mortality rates were significantly lower with BCESs compared to Non-BCESs (1.7 vs. 2.2%). Similarly, at the institute level, AIBCESs exhibited significantly lower rates of anastomotic leakage (12.9 vs. 15.1%) and overall surgery-related mortality (1.6 vs. 2.8%) compared to Non-AIBCESs. Hierarchical logistic regression analysis highlighted that patients treated at AIBCESs had significantly lower surgery-related mortality rates. The institute’s certification had greater influence on short-term surgical outcomes than the operating surgeon’s certification. Regarding long-term outcomes, Motoyama et al. [23] also reported 5-year survival information using the National Database of Hospital-based Cancer Registries. After propensity score matching, significant differences in 5-year survival rates were observed between patients with cStages I–III disease treated at AIBCESs and Non-AIBCESs. Cox proportional hazard regression analyses further demonstrated a significantly lower hazard ratio for patients treated at AIBCESs compared to Non-AIBCESs, indicating a survival advantage associated with undergoing esophagectomy at an AIBCES.

#### Surgeon/hospital volume and mortality

Nishigori et al. [24] investigated the relationship between surgeon/hospital volume and risk-adjusted mortality following esophagectomy in Japan, utilizing the NCD. The same hospital volume categories showed no differences in risk-adjusted operative mortality between surgeon volume categories. Mortality rates in low-volume hospitals (4.8%) were about 2.5 times higher than those in high-volume hospitals with low-volume (1.9%) and high-volume (1.8%) surgeons. In Japan, high-volume hospitals had lower risk-adjusted 30-day and operative mortality rates following esophagectomy compared to low-volume hospitals.

#### Institutional department profiles and mortality

Konno et al. [25] examined the association between institutional department profiles and operative outcomes, using an online questionnaire survey over institutions registered in NCD system. Ten questionnaire items related to surgical indications, patient safety measures, and quality improvement initiatives were identified as positively associated with operative outcomes. Meeting nine or more conditions significantly decreased the odds of operative mortality compared to meeting eight or fewer conditions in all 8 major surgical procedures including esophagectomy. In addition, institutional volume was found to impact operative outcomes, emphasizing the importance of departmental structural and procedural characteristics in patient care.

The NCD conducted a questionnaire survey regarding the significance of board-certified surgeons in cancer treatment and guideline implementation in institutional departments. Toh et al. [26] reported that medical institutional structures, such as AIBCESs and participation of BCESs, improved operative mortality after esophagectomy. Establishing appropriate quality indicators and ensuring their consistent implementation were identified as crucial for enhancing medical care quality in esophagectomy.

#### Hospital centralization and mortality

Takahashi et al. [27] evaluated the impact of centralization on healthcare quality before and after policy restructuring in Japan. In centralized prefectures, the number of esophagectomies performed per hospital increased while the crude operative mortality decreased over time, indicating a positive effect of centralization on healthcare quality.

### Other NCD-related projects

The JSGS collaborates with affiliated societies to collect and analyze data on gastroenterological surgeries. The JES conducts various clinical studies on esophagectomy using the NCD to contribute to improving the diagnosis and treatment of esophageal cancer in Japan.

#### Minimally invasive esophagectomy (MIE) vs. open esophagectomy (OE)

Takeuchi et al. [28] compared short-term outcomes of MIE with OE for thoracic esophageal cancer using NCD from 2011 to 2012. They found that MIE was associated with a lower incidence of prolonged postoperative respiratory ventilation compared to OE, but a higher reoperation rate within 30 days. There were no significant differences between the MIE and OE groups in 30-day mortality rates (0.9 vs. 1.1%) and operative mortality rates (2.5 vs. 2.8%, respectively).

Using data from 2012 to 2016, Yoshida et al. [29] reported that MIE was superior or equivalent to OE in terms of postoperative morbidities and surgery-related mortality, regardless of preoperative treatment type. The total surgery-related mortality rates of MIE and OE were 1.7% and 2.4%, respectively (*P* < 0.001). The results suggest that MIE can replace OE in various situations from the perspective of short-term outcome.

#### Laparoscopy vs. open laparotomy

Takeuchi et al. [30] compared postoperative complications, particularly pulmonary complications, between laparoscopy and open laparotomy for minimally invasive thoracoscopic esophagectomy. They found no significant difference in pulmonary complication incidence between the two groups after matching (20.8 vs. 22.0%). No difference in the incidence of pulmonary complications was observed among patients treated in two different styles of the laparoscopic approach (20.8% in the laparoscopic-assisted surgery group vs. 20.4% in the hand-assisted laparoscopic surgery group).

#### Patient position

Okamura et al. [31] investigated the impact of patient position on postoperative pneumonia occurrence. They found no significant differences in pneumonia incidence between the left lateral decubitus position and the prone position, but noted higher rates of prolonged ventilation and surgery-related mortality in the former.

#### Reconstruction route

Kikuchi et al. [32] investigated the effect of reconstruction route on short-term outcomes after esophagectomy followed by gastric conduit reconstruction. They found significantly lower rates of anastomotic leakage and surgical site infection in the posterior mediastinal group compared to the retrosternal group (11.7 vs. 13.8%, and 8.4 vs. 14.9%, respectively), while the incidence of pneumonia was higher in the former group (13.7 vs. 12.2%).

#### Esophagectomy in elderly patients

Murakami et al. [33] identified risk factors for operative mortality after esophagectomy in elderly (≥ 75 years) patients. They found that elderly patients with residual tumors had significantly higher operative mortality rates compare to increased to non-elderly groups (< 65 or 65–74) (15.9 vs. 5.5 or 6.5%) and much higher than that in elderly patients without residual tumors (15.9 vs. 4.6%). They highlighted the importance of careful selection for the treatment of elderly patients with highly advanced tumors (N2–N3 and M1) to avoid unfavorable short-term outcomes.

#### Preoperative hemoglobin A1c (HbAlc) levels

Okamura et al. [34] investigated the association between preoperative hemoglobin A1c (HbAlc) levels and short-term outcomes after oncologic esophagectomy. They found value-dependent associations between HbA1c values and odds ratios for anastomotic leakage, surgical site infections, pneumonia, and composite outcomes, emphasizing the importance of preoperative glycemic control.

#### Cervical esophageal carcinoma

Nakajima et al. [35] analyzed the surgical outcomes of cervical esophageal carcinoma to evaluate the impact of larynx-preserving surgery. They found no significant differences in morbidity between larynx-preserved and laryngectomy groups. The incidence of postoperative complications related to reconstructed organs was significantly higher in the gastric tube reconstruction than in the free jejunum for anastomotic leakage or pneumonia (17.9 vs. 6.7%, 16.7 vs. 11.1%, respectively), but not different for other complications [36] .

### Studies based on comprehensive registry of esophageal cancer in Japan (CRECJ)

The JES has collected the data on patient characteristics, treatments, and long-term outcomes annually. Oshikiri et al. [37] analyzed data from the CRECJ to assess the impact of routine thoracic duct resection on the prognosis of esophageal cancer patients after radical esophagectomy. They concluded that prophylactic thoracic duct resection does not improve survival in patients with esophageal cancer. Even for those treated with neoadjuvant chemoradiotherapy followed by esophagectomy, thoracic duct resection did not contribute to improved survival [38]. Watanabe et al. reported annual CRECJ implemented to the NCD from 2013 to 2015 [13–15]. These reports, which encompass long-term outcomes, are expected to significantly contribute to the enhancement of all facets related to the diagnosis and treatment of esophageal cancer in Japan.

### JES-certified multi-institutional studies

The JES conducts various questionnaire-based retrospective clinical reviews of esophageal cancer patients who underwent surgical treatment, collaborating with authorized institutions (AIBCES) certified by JES.

#### Tumor marker

Suzuki et al. [39] evaluated the clinical significance of preoperative serum p53 antibodies status in esophageal cancer patients. They found an association between p53 antibody presence and tumor progression, but it was not an independent risk factor for poor prognosis. They also identified carcinoembryonic antigen (CEA) [40] and serum C-terminus of cytokeratin 19 (CYFRA) [41] as prognostic determinants in esophageal squamous cell carcinoma (ESCC) patients.

#### Surgical resection of recurrent lesions

Kudou et al. [42] identified a subset of patients who benefit from surgical resection of recurrent lesions after curative esophagectomy for esophageal squamous cell carcinoma, such as cases involving pN 0–1, lung metastasis, recurrence-free interval after curative esophagectomy of ≥ 550 days, and technically resectable lesions.

#### Chemoradiotherapy with complete response diagnoses

Mori et al. [43] investigated outcomes of primary thoracic esophageal cancer patients initially treated by chemoradiotherapy with complete response diagnoses. They found that a quarter of patients developed recurrent disease, mostly locoregional, after complete response. Salvage treatments achieved modest long-term survival.

#### Gastric tube cancer

Ota et al. [44] conducted a retrospective nationwide survey of gastric tube cancer after esophagectomy, emphasizing the importance of early detection through regular upper gastrointestinal endoscopy continuing every 2 years for 10 or more years.

#### Neoadjuvant chemotherapy and recurrence

Matsuda et al. [45] assessed the effectiveness of neoadjuvant docetaxel, cisplatin, and 5-fluorouracil (DCF) therapy compared to cisplatin and 5-fluorouracil (CF) in patients with surgically resectable advanced ESCC, demonstrating a survival advantage with neoadjuvant DCF therapy particularly in patients who were 75 years old or younger. In addition, they validated the prognostic significance of stratification based on pathological stage and response to neoadjuvant chemotherapy [46]. Furthermore, they investigated the impact of regional recurrence on survival relative to the magnitude of pathological response [47]. Notably, a pathological complete response (pCR) in ESCC patients with cT1/2 was associated with a favorable prognosis and reduced incidence of distant failure, suggesting its potential utility in selecting appropriate candidates for organ preservation approaches based on induction therapy response. The prognostic value of postoperative complications was evaluated by stratifying patients based on age and neoadjuvant chemotherapy regimen [48], with results confirming a negative impact on survival. Moreover, the adverse effects were more pronounced in elderly patients receiving triplet chemotherapy. In addition, the prognostic impact of endoscopic response (ER) was assessed and validated [49], highlighting the necessity for standardizing ER evaluation to enhance interinstitutional consistency and clinical validity. Furthermore, Okamura et al. developed and validated models predicting recurrence of ESCC [50] and therapeutic effects [51] in patients undergoing neoadjuvant treatment prior to esophagectomy.

### Retrospective cohort studies using DPC database

The DPC data include administrative claims data, such as anesthesia, surgery, rehabilitation and other procedures, pharmaceuticals and devices used. The database includes estimated total costs based on reference prices in the Japanese national fee schedule that determine item-by-item prices for surgical, pharmaceutical, laboratory, and other inpatient services [16]. The DPC with its advantages in data comprehensiveness and validity (e.g., various measures of severity, distinguishability between co-morbidity presenting on admission and complications occurring during hospitalization, as well as numerous records regarding procedures and medications) has been utilized in numerous studies involving large patient cohorts [52].

Sakamoto et al. [53] compared surgical outcomes of MIE and OE for esophageal cancer from 2014 to 2017, demonstrating favorable outcomes with MIE in terms of mortality, morbidities, and postoperative hospital stay.

Hirano et al. investigated compared the impact of epidural analgesia (EDA) [54] on short-term outcomes in MIE patients, as well as the effect of prophylactic corticosteroid [55] and antimicrobial prophylaxis [56] on postoperative outcomes. EDA use was associated with low in-hospital mortality, as well as decreased respiratory complications and anastomotic leakage. The prophylactic corticosteroid use resulted in lower in-hospital mortality and decreased respiratory failure. The administration of ampicillin-sulbactam (ABPC/SBT) as antimicrobial prophylaxis was associated with better short-term postoperative outcomes compared to cefazolin (CEZ). Furthermore, weight loss during neoadjuvant therapy (NAT) was associated with failure to rescue (the proportion of mortality in patients with at least one major complication) and in-hospital mortality [57], emphasizing the importance of weight loss measurement during NAT to assess the risk for a subsequent esophagectomy.

## Conclusions

The feedback system of NCD provides the predicted mortality and morbidity of patient with preoperative data input, and each facility’s severity-adjusted clinical performance compared with the national data. Certification systems established by JSGS and JES have demonstrated the greater influence of institutional certification on short-term surgical outcomes than that of operating surgeon’s certification. Minimally invasive esophagectomy has shown promise as an alternative to open esophagectomy, with careful patient selection being crucial, particularly for elderly patients with advanced tumors.

Research utilizing nationwide databases such as NCD, CRECJ, and DPC has significantly advanced our understanding of esophagectomy outcomes and contributed to quality improvement in healthcare. Ongoing studies aim to further improve the quality control of esophagectomy.

## Data Availability

Data availability is not applicable to this article as no new data were created or analyzed in this study.
